# Tumor mutational burden as a predictor of immunotherapy response in breast cancer

**DOI:** 10.18632/oncotarget.27877

**Published:** 2021-03-02

**Authors:** Tess A. O’Meara, Sara M. Tolaney

**Affiliations:** ^1^Department of Internal Medicine, Brigham and Women’s Hospital, Boston, MA, USA; ^2^Department of Medical Oncology, Dana-Farber Cancer Institute, Boston, MA, USA

**Keywords:** breast cancer, immunotherapy, tumor mutational burden

## Abstract

Tumor mutational burden (TMB) is a promising tool to help define patients with triple-negative breast cancer (TNBC) most likely to benefit from immune checkpoint blockade (ICB) therapies. Roughly reflecting the degree of neo-antigens that tumors present to immune cells, TMB associates with multiple measures of tumoral immunogenicity and has proven clinically useful in cancers with relatively high mutation burden. TNBC carries higher TMB than other breast cancer subtypes, and recent data suggest that high-TMB TNBC cases may derive particular benefit from ICB in combination with chemotherapy (GeparNuevo, IMpassion130) or even ICB alone (KEYNOTE-119, TAPUR). Given the recent approval of pembrolizumab and atezolizumab in combination with chemotherapy for PD-L1-positive, metastatic TNBC, standardizing TMB calculation methods and cut-off values is of critical importance to deploy this clinical biomarker.

## INTRODUCTION

Defining patients most likely to derive durable benefit from immune checkpoint blockade (ICB) is critical to the progress of immuno-oncology. Recent approval of both pembrolizumab and atezolizumab in combination with standard chemotherapy for programmed cell death 1 ligand 1 (PD-L1)-positive, metastatic triple-negative breast cancer (TNBC) represents an important step forward for the use of ICB in breast cancer [1, 2]. However, PD-L1 expression has technical limitations as a biomarker due to its dynamic and heterogeneous expression in the tumor microenvironment, variable assay interpretation, and a lack of standardization across platforms [3–5]. Pembrolizumab has now been approved for metastatic TNBC with combined positive score (CPS) ≥ 10 using the 22C3 assay, while atezolizumab carries approval for immune cell score (IC) ≥ 1 with SP142. Additionally, only a fraction of patients with PD-L1-positive, metastatic TNBC respond to ICB [6, 7]. DNA instability has emerged as a predictor of response to ICB alone and in combination with chemotherapy and DNA-damaging agents [8]. These observations culminated in the U.S. Food and Drug Administration (FDA) pan-approval of pembrolizumab for unresectable or metastatic cancers of any tissue origin with microsatellite instability (MSI+) or mismatch repair deficiency (MMR-) [9].

Although TNBC generally carries higher mutation rates than other breast cancer subtypes, less than 2% of all breast cancer cases are classifiable as MSI+, suggesting that TNBC may accumulate mutations through alternate pathways [10]. Tumor mutational burden (TMB), more generally quantifying the number of non-synonymous somatic mutations, also predicts benefit to ICB in melanoma, lung, urothelial, and colon cancer [11–14]. Whereas other biomarkers of ICB response, including PD-L1 expression, T-cell inflammation signatures, and tumor infiltrating lymphocyte (TIL) counts, directly measure immune activity, TMB indirectly reflects tumor immunogenicity by approximating neoantigens presented to major histocompatibility complexes (MHC). Therefore, we address the question: can TMB be utilized as a biomarker of immunotherapy response in metastatic TNBC?

Breast cancer carries an intermediate TMB compared to cancers in which immunotherapy is widely used. Studies of large cancer databases have reported a median of 2.63 mutations per genomic megabase (mut/MB) among all breast cancers, compared to 7.2 mut/MB in lung cancer and 13.5 mut/MB in melanoma [15, 16]. In general, cancers with high TMB also carry higher TIL counts, higher expression of immune gene signatures, and substantial survival benefits from anti-PD1 therapies. Using a common definition of TMB-high as ≥ 10 mut/MB, approximately 5% of all breast cancer cases (including all breast cancer subtypes) are considered TMB-high, compared to 4% TIL-predominant (defined by ≥ 50% lymphocyte infiltration) and 10% PD-L1-positive (defined by ≥ 1% tumor or immune cell expression) [7, 15, 17, 18]. Approximately 40% of metastatic TNBC are PD-L1-positive [1, 2].

Nonetheless, among breast cancer cases, TMB varies greatly with histopathologic and clinical variables. TNBC, particularly of basal subtype, carries higher TMB than estrogen receptor (ER)-positive or HER2-positive breast cancers [19, 20]. Consistent with this observation, PD-L1-positive TNBC also is associated with higher TIL counts and higher response rates to anti-PD1 therapies [21]. Evidence suggests that lobular carcinomas contain higher TMB than ductal [15]. Beyond the mutational load, differences in mutational signatures are thought to influence immunogenicity as well. For example, carcinogen-related DNA damage, dominated by C>A transversions, is thought to be most predictive of ICB response [22] in melanoma and lung cancer. Tumors associated with *BRCA1* and *BRCA2* germline mutations, which are disproportionately TNBC, acquire distinctly high mutational loads through homologous recombination deficiency and contain high PD-L1 expression [23]. Evidence has suggested that the APOBEC mutation signature, associated with cytidine deaminase dysregulation, may be the driving mutational process in breast cancer, dominant in more than half of hypermutated cases [15]. Durable response to ICB not achieved with standard chemotherapy has been reported in high-TMB breast cancer with APOBEC signature, despite low TIL counts and PD-L1 negatively [24]. Notably, the approval of pembrolizumab for MMR- or MSI+ cancers would miss a substantial number of these cases. Only 0.04–1.53% of all breast cancers are classifiable as MSI+, while approximately 33–46% of metastatic breast cancers demonstrate APOBEC mutagenesis [25–27]. In June, 2020, based on results from phase II KEYNOTE-158, pembrolizumab was FDA approved for non-MMR-/MSI+ TMB-high previously treated, advanced solid tumors [28]. Breast cancer was not included in this study; eligible tumor types were anal, biliary, cervical, endometrial, mesothelioma, neuroendocrine, salivary, small-cell lung, thyroid, and vulvar.

Metastatic breast cancers also contain higher TMB than primary cancers (estimated 8–11% versus 2–5% TMB-high, respectively), which may be related to the tendency of more genomically unstable cells to metastasize or the acquisition of additional mutations over time [7, 15, 24]. Importantly, there does not seem to be an association between TMB and the risk of primary tumor metastasis [15]. In fact, high TMB has been associated with prolonged overall survival (OS) in de novo, treatment-naïve metastatic TNBC [29]. Potentially as a result of up-regulation of immune-escape mechanisms and complex clonal pruning, metastatic cancers are known to be less immunogenic than their primary counterparts, including relatively lower PD-L1 expression [30–32]. While the established benefit of ICB in metastatic TNBC has led to several FDA approvals, there is increasing evidence that ICB also has benefit in early-stage disease, potentially independent of PD-L1 expression [33]. This may be attributable to the more active immune microenvironment in early-stage tumors. Analyses of recent neoadjuvant trial data from GeparNuevo suggest that TMB may have clinical utility in predicting pathologic complete response (pCR) from ICB in early-stage TNBC as well [34]. This analysis included 149 early-stage TNBC receiving neoadjuvant durvalumab and chemotherapy (*n* = 74) or chemotherapy alone (*n* = 75). Median TMB was significantly higher in patients with pCR (median 1.87 versus 1.39, *p* = 0.005), and odds ratios for pCR per mut/MB were 2.06 (95% confidence intervals [CI] 1.33–3.20) among all patients, 1.77 (95% CI 1.00–3.13) in the durvalumab arm, and 2.82 (95% CI 1.21–6.54) in the chemotherapy arm. Although the association between pCR and TMB was present in both the ICB dual therapy and chemotherapy arms, it was stronger in the cohort treated with chemotherapy alone.

In contrast, in the metastatic setting, multiple exploratory, retrospective analyses of recent clinical trials support the benefit of ICB monotherapy in TMB-high TNBC. The KEYNOTE-119 trial compared pembrolizumab monotherapy to chemotherapy in 622 patients with pre-treated, metastatic TNBC [35]. Of the 253/601 treated patients with available TMB data (*n* = 132 pembrolizumab, *n* = 121 chemotherapy arm), 26 patients (10.3%) were TMB-high (TMB ≥ 10 mut/MB). There was a positive association between TMB and clinical response to pembrolizumab (overall response rate [ORR] *p* = 0.154, progression free survival [PFS] *p* = 0.014, OS *p* = 0.018) but not to chemotherapy (ORR *p* = 0.114, PFS *p* = 0.478, OS *p* = 0.906). In TMB-high cases, ORR and hazard ratio (HR) for OS also suggested a trend towards increased benefit with pembrolizumab versus chemotherapy. Although suggestive for the clinical utility of TMB in pre-treated metastatic TNBC, this study was limited by the limited sample size and low number of TMB-high cases.

These findings were supported by the phase II TAPUR trial, a prospective study evaluating single-agent pembrolizumab in 28 heavily pre-treated metastatic breast cancer of all subtypes [36]. This study detected benefit from ICB monotherapy in TMB-high (TMB ≥ 9 mut/MB) cases with an ORR of 21% (95% CI 8–41%) and PFS rate of 10.6 weeks (95% CI 7.7–21.1 weeks). No association was found between increasing TMB and longer PFS. A recent retrospective analysis of 62 patients with metastatic TNBC also suggests ICB therapy alone may be sufficient to treat TMB-high tumors [37]. Patients in this study had received either anti-PD-1/PD-L1 alone (23%) or in combination with targeted therapy (19%) or chemotherapy (58%), and 60% had received one or more prior therapies for metastatic disease. TMB-high (TMB ≥ 10 mut/MB) cases demonstrated significantly longer PFS (12.5 versus 3.7 months, *p* = 0.03), longer OS (29.2 versus 14.2 months, *p* = 0.06) and a 4 times higher odds of response than patients without high TMB (odds ratio = 4.32, 95% CI 1.05–19.89) in multivariate analyses. The association between PFS and TMB was independent of monotherapy versus combination therapy regimen as well as number of prior treatment lines. Notably, the association was also independent of PD-L1 status.

The results described suggest that ICB monotherapy may be sufficient to treat TMB-high metastatic TNBC as well as relevant for early-line treatment. The rationale to combine chemotherapy with immune-modulating agents dates back more than 20 years; biologically, DNA-damaging chemotherapies as well as targeted therapies such as PARP inhibitors are thought to sensitize tumors to ICB treatment by further disrupting tumor cell DNA repair pathways, increasing TMB, promoting neoantigen expression, and activating the STING pathway [23, 38]. Mutagenesis in hypermutated, metastatic TNBC appears to be driven largely by APOBEC processes and associated with frequent *PIK3CA* mutations [15]. Therefore, pairing ICB with direct AKT inhibitors that target the PIK3CA pathway may provide particular benefit in these cases [39]. Results from a recent phase Ib study of the AKT inhibitor ipatasertib combined with nab-paclitaxel and atezolizumab in 26 patients with metastatic TNBC support this hypothesis, demonstrating an ORR of 73%, independent of *PIK3CA/AKT1/PTEN* mutation status [40]. Other work has suggested that *PIK3CA* mutations are associated with a complete or partial response to ICB in MSI+ solid tumors [41]. However, evidence has emerged to suggest that chemotherapy does not always confer additional benefit and is associated with additional toxicity [42]. In some cases, priming with chemotherapy may even promote the expansion of less immunogenic subclones, reducing ICB effectiveness [43]. Lead-in therapy with ICB may an alternative, more effective strategy. In the phase II GeparNuevo study, patients with TNBC who received durvalumab monotherapy prior to ICB and chemotherapy together were more likely to obtain pCR than those who received the combination alone [44]. First-line, single agent ICB therapies are approved in melanoma (ipilimumab, nivolumab, or pembrolizumab), non-small cell lung cancer (pembrolizumab) and locally advanced urothelial cancer (pembrolizumab, atezolizumab), three cancers with among the highest TMB [45]. TMB-high, metastatic TNBC may benefit from similar approval. Dual ICB therapy may be another strategy to maximize benefit and minimize toxicity; to evaluate this hypothesis, we have launched a multicenter, single arm, phase II trial (NIMBUS) of nivolumab plus ipilimumab in metastatic, hypermutated HER2-negative breast cancer (NCT03789110).

The relationship between the predictive values of PD-L1 expression and TMB is of great debate. Several studies have shown that PD-L1 and TMB are independent predictors of ICB response, and PD-L1 expression and TMB have low correlation across multiple tumor types [46, 47]. However, the landmark IMpassion130 trial, investigating nab-paclitaxel monotherapy versus atezolizumab plus nab-paclitaxel in previously untreated, metastatic or locally advanced TNBC, found that TMB predicted increased benefit to ICB only in PD-L1-positive tumors [1]. In this study of 579 patients, increasing TMB was associated with improved PFS (highest TMB quartile HR 0.56, 95% CI 0.38–0.81), but the association was primarily driven by the PD-L1-positive subgroup (HR 0.31 versus 0.84). Increasing TMB was also associated with prolonged OS in the PD-L1-positive group alone. It is possible that an algorithm integrating T-cell inflammation, PD-L1 expression, and TMB biomarkers will best identify patients most likely to benefit from ICB monotherapy or combination regimens [48]. Other studies have suggested ICB response in TMB-high tumors may not be dependent on PD-L1 expression in the setting of anti-CTLA4 or anti-PD-1/CTLA-4 combination therapy [49]. Due to the limitations of PD-L1 as a biomarker described above, establishing the independent benefit of TMB in predicting response to both anti-PD-1/PD-L1 and anti-CTLA4 therapies would be a highly useful clinical tool.

A major barrier to the incorporation of TMB into clinical practice is standardizing the methods of TMB measurement. Although the initial reports showing correlation between TMB and immunotherapy response were performed using whole-exome sequencing (WES), many studies have proven the feasibility of predicting TMB on lower-cost, targeted gene panels [16, 50]. However, several challenges still remain, including defining the “TMB-high” threshold, which varies widely by tumor type, selecting how many and which genes to include, standardizing the mutation calling pipeline, and accurately removing germline variants. Evidence has shown more discordance between WES versus targeted panel estimates in tumors with moderate to low underlying TMB [51]. Moreover, although many clinical trials utilize the FoundationOne targeted gene panel, establishing interoperability between panels of different sizes is an important step. Measuring TMB on peripheral blood provides a unique opportunity to reduce invasive procedures for patients and re-test TMB over the course of treatment. Although circulating tumor DNA (ctDNA) is not available in all cases and may reflect metastatic disease over primary tumor biology, TMB values from WES of ctDNA have proven consistent with those from WES on tissue biopsies [52].

In conclusion, there is great potential for TMB to define a subset of patients with metastatic TNBC most likely to benefit from ICB. With additional investigation, TMB may prove to be useful for predicting ICB response in early-stage disease as well. Defining the means to measure TMB accurately, reproducibly and cost-effectively is an important task for the implementation of this promising clinical biomarker.
